# Bioactive Compounds from Macroalgae in the New Millennium: Implications for Neurodegenerative Diseases

**DOI:** 10.3390/md12094934

**Published:** 2014-09-25

**Authors:** Mariana Barbosa, Patrícia Valentão, Paula B. Andrade

**Affiliations:** REQUIMTE/Laboratory of Pharmacognosy, Department of Chemistry, Faculty of Pharmacy, University of Porto, Rua de Jorge Viterbo Ferreira n° 228, 4050-313 Porto, Portugal; E-Mails: mariana.nunes.barbosa@gmail.com (M.B.); valentao@ff.up.pt (P.V.)

**Keywords:** macroalgae, neuroprotection, neurodegeneration, neuroinflammation, oxidative/nitrosative damage, cholinesterase, Alzheimer’s, Parkinson’s

## Abstract

Marine environment has proven to be a rich source of structurally diverse and complex compounds exhibiting numerous interesting biological effects. Macroalgae are currently being explored as novel and sustainable sources of bioactive compounds for both pharmaceutical and nutraceutical applications. Given the increasing prevalence of different forms of dementia, researchers have been focusing their attention on the discovery and development of new compounds from macroalgae for potential application in neuroprotection. Neuroprotection involves multiple and complex mechanisms, which are deeply related. Therefore, compounds exerting neuroprotective effects through different pathways could present viable approaches in the management of neurodegenerative diseases, such as Alzheimer’s and Parkinson’s. In fact, several studies had already provided promising insights into the neuroprotective effects of a series of compounds isolated from different macroalgae species. This review will focus on compounds from macroalgae that exhibit neuroprotective effects and their potential application to treat and/or prevent neurodegenerative diseases.

## 1. Introduction

Oceans account for 71% of the earth’s surface and are the largest remaining reservoirs of bioactive compounds [1]. The ﬁrst serious effort in studying marine natural products started in 1951 with the pioneering work of Bergman and Feeney that resulted on the isolation of spongothymidine and spongouridine from the sponge *Cryptotethya crypta* Laubenfels [2]. This finding led to the synthesis of arabinosyl cytosine (Ara-C), a marine-derived anticancer agent used mainly in the treatment of different forms of leukemia. Since the 1950s, it has been shown that marine organisms are rich sources of structurally novel and biologically active metabolites, constituting valuable opportunities for drug discovery, an area of extreme importance among the scientific community [3].

Macroalgae are abundant and potentially renewable resources that are currently being explored as novel and sustainable sources of compounds for both pharmaceutical and nutraceutical applications [4]. They can be classified according to the presence of specific pigments into Chlorophyceae (green algae), Phaeophyceae (brown algae) and Rhodophyceae (red algae) [5]. The color of Chlorophyta is due to the presence of chlorophylls *a* and *b* in the same proportions as in terrestrial higher plants. The greenish brown color of Phaeophyta is attributed to the presence of fucoxanthin, chlorophylls *a* and *c*. Phycobilins, such as phycoerythrin and phycocyanin, are responsible for the color of Rhodophyta [6].

Marine algae have a long tradition as a food source in Asian countries, being part of the Western diet only to a limited extent. In 2003, the total annual value of global seaweed production was estimated to be almost US$6 billion, of which food products for human consumption represented US$5 billion [7]. Although global marine algae utilization is a multibillion dollar industry, their bioactive potential is still underexploited. For centuries, the medicinal properties of macroalgae were limited to traditional and folk medicines [8]. However, in recent years, industries from different branches (fuel, varnish, textile, paints, plastics, cosmetics, pharmaceutical and food) have been focusing their attention on the discovery and development of compounds from marine algae [9].

The great diversity in terms of the number of different species of macroalgae, combined with the hostile and extreme conditions of light, salinity and temperature in which some species inhabit, can explain the growing interest in the discovery and isolation of primary and secondary metabolites produced by these unique organisms [10].

Several studies have shown that secondary and some primary metabolites from green, brown and red marine algae exhibit numerous promising and remarkable biological activities, providing important chemical scaffolds for the discovery of new drugs for the management of some chronic diseases [11]. For instance, dieckol, a phlorotannin isolated from *Ecklonia cava* Kjellman was revealed to play an important role in the prevention of type ІІ diabetes [12]; aplysistatin, a brominated sesquiterpene with high degree of unsaturation isolated from *Laurencia snackeyi* (Weber-van Bosse) Masuda was able to modulate specific agents involved in inflammatory response [13]; fucoxanthin, one of the most abundant carotenoids found in brown algae, exhibited preventive effects on cancer through antioxidant, antiproliferative and anti-angiogenic mechanisms [14].

## 2. Neurodegenerative Diseases: Alzheimer’s and Parkinson’s

Neurodegenerative diseases are estimated to surpass cancer as the second most common cause of death among elderly by the 2040s [15]. Therefore, during the past decade, the neuroprotective effects of different compounds from marine algae have been investigated in order to be potentially applied in the management of neurodegenerative diseases, such as Alzheimer’s (AD) and Parkinson’s (PD) [16].

Dementia is an umbrella term used for chronic progressive mental disorders affecting memory, thinking, comprehension and other essential brain functions. AD and PD are the most common types of dementia, their prevalence being dramatically increasing worldwide [17].

AD is an irreversible and progressive neurodegenerative disease characterized by memory loss, behavior disturbances, personality changes and decline of cognitive abilities. This common form of dementia is presently the sixth-leading cause of death [18]. The main pathological hallmarks of AD are the formation of senile plaques and neurofibrillary tangles. However, the most remarkable biochemical change in AD patients is the reduction of acetylcholine (ACh) levels in the hippocampus and cortex of the brain [19,20].

PD is a multidimensional progressive disease with a number of motor and non-motor features, including cognitive dysfunction [21]. It is estimated that 6.3 million people worldwide have PD. This disease is neuropathologically characterized by the aggregation and accumulation of α-synuclein protein of Lewy bodies (LB) and loss of dopaminergic neurons in the substantia nigra (an area in the basal ganglia), resulting in a significant reduction of dopamine content in the striatum and corresponding loss of dopamine transporters [22,23].

### 2.1. Mechanisms of Neurodegeneration 

Neurodegenerative diseases are associated with numerous and complex phenomena, such as neuroinflammation, extensive oxidative/nitrosative damage caused by reactive oxygen and nitrogen species (ROS and RNS), respectively, synaptic loss and other potential pathways of neuronal cell death [24,25,26]. 

#### 2.1.1. Neuroinflammation

Recently, studies have shown that activation of microglia cells, mediators of the innate responses in the central nervous system (CNS), and the resulting production of pro-inflammatory and neurotoxic factors like nitric oxide (^•^NO), prostaglandin E2 (PGE2), ROS, interleukin (IL)-1β, IL-6 and tumor necrosis factor (TNF)-α can induce neurodegeneration [25,27,28]. The release of excessive amounts of pro-inflammatory mediators by microglia has been observed during the pathogenesis of PD and AD. Therefore, mechanisms to regulate microglial activation may potentially reduce neuronal injury or death in neurodegenerative diseases [29]. Indeed, epidemiological studies have already shown that long-term treatment with non-steroidal anti-inflammatory drugs (NSAIDs) reduces the risk of AD, delays the disease’s onset, ameliorates symptomatic severity and slows cognitive decline [30]. However, the widespread use of NSAIDs increases the risk of developing gastrointestinal and kidney problems. These side effects have stimulated the search for alternative anti-inflammatory drugs from natural renewable sources, such as macroalgae. Recent works have revealed that marine compounds can modulate signaling pathways like c-Jun *N*-terminal kinase (JNK), mitogen-activated protein kinase (MAPK) and Akt/protein kinase B (PKB) pathways, which are connected to the regulation of the neuroinflammatory response [31,32].

#### 2.1.2. Oxidative/Nitrosative Damage

Several studies have established that the imbalance between pro-oxidant and antioxidant homeostasis, leading to the generation of toxic ROS and RNS, may be involved in the pathogenesis of most of the neurodegenerative disorders [33,34]. The accumulation of ROS and RNS and the interaction between these reactive species can result in lipid peroxidation, protein oxidation, DNA damage and, ultimately, in neuronal cell death [34,35]. Therefore, exogenous antioxidants may have positive effects in the removal or suppression of the generation of ROS/RNS, thus preventing neuronal cell death. A large number of potent antioxidant compounds has already been detected in different macroalgae, including phlorotannins, sulfated polysaccharides, carotenoids and sterols, making these marine organisms valuable sources of compounds with neuroprotective effects [35,36,37,38]. Emerging evidences suggest that antioxidant activity cannot be the exclusive mechanism by which compounds exert neuroprotection, but, rather, their ability to potentially alter signaling pathways involved in cell survival systems [39].

#### 2.1.3. Synaptic Loss

A common pathological hallmark of various neurodegenerative diseases is the loss of particular subsets of neurons [37]. Although cholinergic denervation is recognized as a pathological hallmark of AD, *in vivo* neuroimaging studies revealed the loss of cerebral cholinergic markers in parkinsonian dementia, similar to or more severe than in prototypical AD [40]. Therefore, a decline of ACh levels is observed in both neurodegenerative disorders. Two types of cholinesterase (ChE) are found in the CNS: acetylcholinesterase (AChE) and butyrylcholinesterase (BuChE). AChE is a substrate-specific enzyme, which degrades ACh in cholinergic synapses, while BuChE is a non-specific enzyme expressed in neuroglia and found in the intestine, liver, kidney, heart, lung and serum. Both enzymes are able to cleave more than 10,000 molecules of ACh *per* second presenting valuable therapeutic targets in neurodegenerative diseases [41,42]. ChE inhibitors retard the inactivation of ACh after synaptic release, representing one of the most realistic and effective approaches to the symptomatic treatment of neurodegenerative disorders [43]. Studies have already shown that ChE inhibitors not only increase the levels of ACh in the brain, but also reduce and prevent the formation of β-amyloid (Aβ) deposits, protecting neurons from neurodegeneration [44].

**Figure 1:**
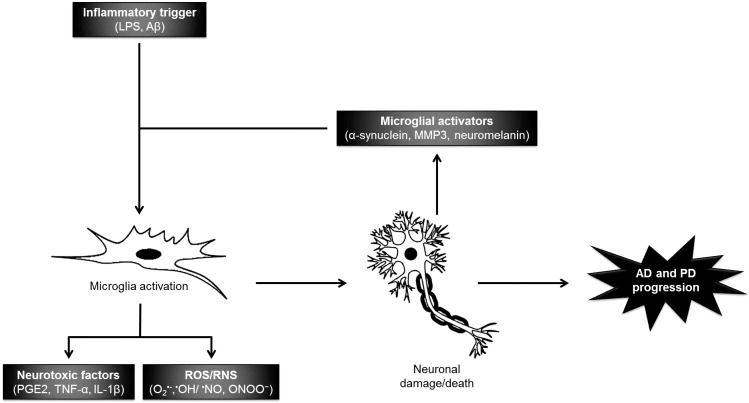
Microglia-mediated neurotoxicity in Alzheimer’s Disease (AD) and Parkinson’s Disease (PD).

The mechanisms previously described are deeply related, as can be observed in Figure 1. In the case of AD, the presence of Aβ deposits has neurotoxic effects by inducing the generation of pro-inflammatory cytokines, ROS and RNS, leading to neuronal dysfunction and eventually cell death [45,46,47]. In PD, the release and accumulation of LB and α-synuclein aggregates induce the activation of microglial cells, resulting in the production of pro-inflammatory mediators, which can also lead to neuronal death [23,48]. 

Oxidative and nitrosative stress markers are also important in neurodegeneration and progression of both AD and PD, leading to the activation of surrounding glia and development of a robust glia-mediated inflammatory response.

## 3. Neuroprotective Compounds from Macroalgae

### 3.1. Phlorotannins

**Figure 2:**
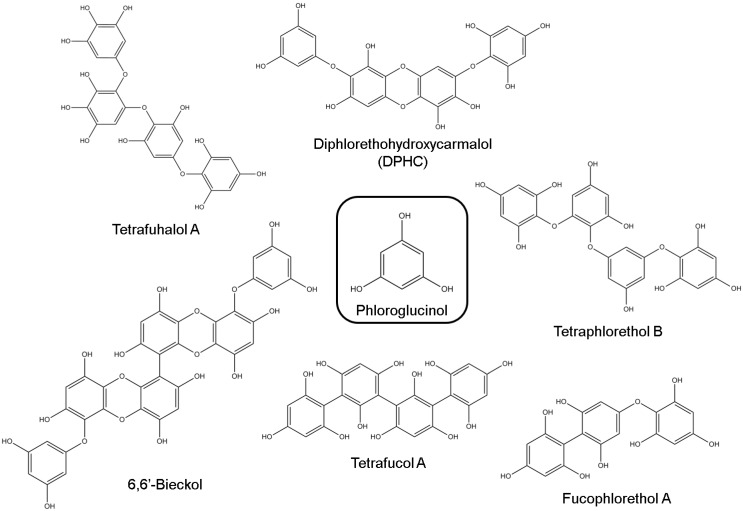
Chemical structures of different types of phlorotannins.

Among several classes of algal polyphenols, phlorotannins are pharmacologically prominent compounds. They are composed of several phloroglucinol (1,3,5-trihydroxybenzene) units, linked to each other by different ways [49]. According to the nature of the structural linkages between phloroglucinol units and the number of hydroxyl groups present, phlorotannins can be subdivided into six specific groups: phlorethols, fuhalols, fucols, fucophlorethols, eckols and carmalols (Figure 2) [50].

These compounds are biosynthesized *via* acetate-malonate pathway and released in response to cellular damage [51]. The distribution of phlorotannins in nature is limited to brown algae and their amounts can vary among species, being affected by algae size, age, tissue type, salinity, season, nutrient levels, intensity of herbivory, light intensity and water temperature [52]. As other polyphenolic compounds, phlorotannins exhibit numerous remarkable properties on biological systems, namely antioxidant [53], anti-inflammatory [54], anti-allergic [55], antimicrobial [56], anticancer [57] and antidiabetic [58] activities. Moreover, phlorotannins also display an important role in neuroprotection through different action mechanisms.

Dieckol and phlorofucofuroeckol, phlorotannins present in *E. cava*, are related with the increment of major central neurotransmitters in the brain of a selected animal model, particularly of ACh, by inhibiting the activity of AChE [59].

**Figure 3:**
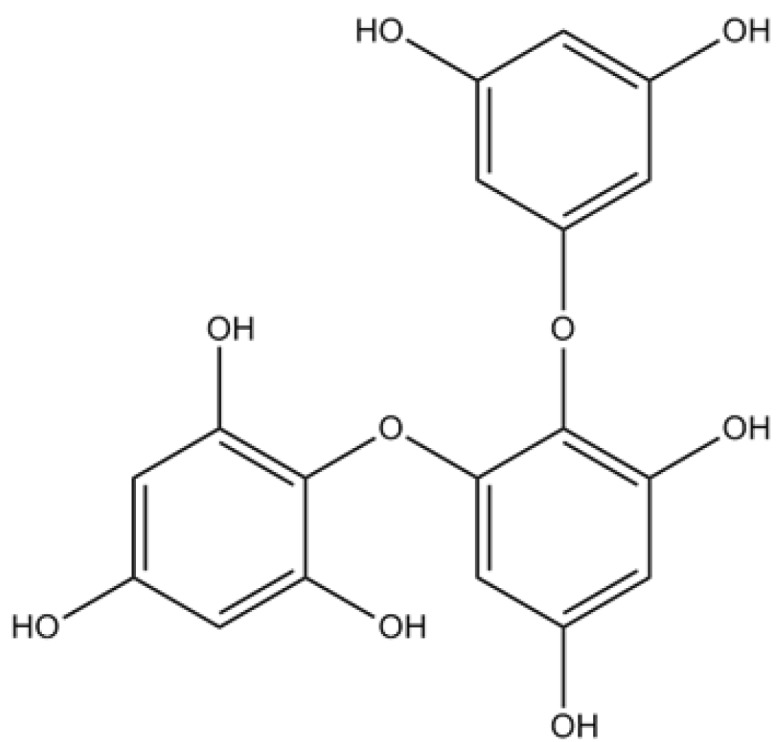
Chemical structure of triphlorethol A.

Eckol, dieckol, 2-phloroeckol and 7-phloroeckol isolated from *Ecklonia stolonifera* Okamura, a perennial brown algae widely distributed throughout the Eastern and Southern coasts of Korea, exhibited a selective dose-dependent inhibitory activity against AChE; eckstolonol and phlorofucofuroeckol A inhibited both AChE and BuChE. However, neither phloroglucinol, nor triphlorethol A (Figure 3), an opened-chain trimer of phloroglucinol, inhibited ChE at the tested concentrations. These results not only suggested that phlorotannins’ structures prevent the binding of substrates to ChE, but also that the degree of polymerization and closed-ring structure must play key roles in phlorotannins’ potential against ChE [60].

In another work, Yoon *et al*. [61] isolated phloroglucinol, 6,6′-bieckol and diphlorethohydroxycarmalol (DPHC) from *Ishige okamurae* Yendo, a brown edible algae found throughout the upper and middle intertidal zones on rough open coast in Korea. They assessed their ChE inhibitory capacity, demonstrating that 6,6′-bieckol and DPHC displayed potent AChE and moderate BuChE inhibitory effects, respectively. According to Lineweaver-Burk plot studies of enzyme kinetics, 6,6′-bieckol acts as a non-competitive inhibitor [61].

Jung *et al*. [29] evaluated the neuroprotective effects of dieckol isolated from *E. cava* (from Jeju Island, Korea) by studying its anti-inflammatory properties. The results showed that dieckol was able to significantly reduce the expression and the release of pro-inflammatory mediators and cytokines, such as ^•^NO, PGE2, IL-1β and TNF-α through down-regulation of nuclear factor κB (NF-κB), p38 kinase activation and/or inhibition of ROS signal in microglial cells [29]. Therefore, dieckol may have beneficial effects in the management of microglia-mediated oxidative stress and neuroinflammation, which are important for the establishment of neurodegenerative processes.

As already mentioned, neurodegeneration can also be the result of an extensive oxidative/nitrosative damage caused by ROS/RNS. More recently, Heon *et al*. [38] evaluated the neuroprotective potential of DPHC based on its antioxidant capacity. This study revealed that DPHC was able to protect cells from oxidative stress-induced neurotoxicity, which may offer health benefits, including prevention of neurodegenerative diseases [38].

Ahn *et al*. [62] studied the neuroprotective effects of the methanolic extract of *Eisenia bicyclis* (Kjellman) Setchell (purchased from a local telemarketing company in Korea) together with those of the isolated phlorotannins on Aβ-induced toxicity in PC12 cells mediated by the suppression of intracellular ROS and the reduction of Ca^2+^ levels. The authors demonstrated that, among the tested compounds, 7-phloroeckol and phlorofucofuroeckol A were potent neuroprotective agents, while eckol exhibited a weaker effect. These results suggest that the molecular size and the number of hydroxyl groups in phlorotannins’ molecules are important features dictating their neuroprotective effects against Aβ-induced cytotoxicity [62].

Hydrogen peroxide (H_2_O_2_) generation is required to mediate the complete sequence of events occurring in oxidative stress-induced neuronal cell death [63]. Evidences indicate that phlorotannins isolated from *E. cava* (from Jeju Island, Korea), including phloroglucinol, eckol, triphlorethol A, eckstolonol and dieckol, were able to protect murine hippocampal HT22 cells against H_2_O_2_-induced neurotoxicity [35].

The antioxidant activity exhibited by phlorotannins can be the result of specific scavenging of radicals formed during peroxidation, scavenging of oxygen/nitrogen-containing compounds or metal-chelating ability [64].

**Figure 4:**
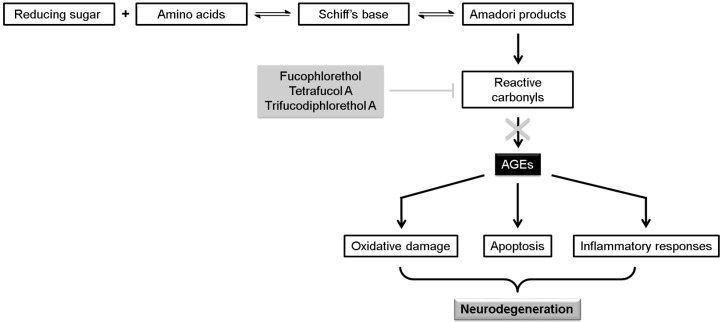
Inhibition of the formation of advanced glycation end products (AGEs) by some phlorotannins through the scavenging of reactive carbonyl intermediates.

The accumulation of advanced glycation end products (AGEs) is being associated with a wide variety of chronic conditions, including neurodegenerative diseases [65]. A number of phlorotannin oligomers, including fucophlorethol A, tetrafucol A and trifucodiphlorethol A, isolated from *Fucus vesiculosus* Linnaeus (obtained from Maine Seaweed Co. (Steuben, ME, USA)), was able to scavenge reactive carbonyls, inhibiting the formation of AGEs (Figure 4) [66].

Although different strategies can be used to prevent the progression of neurodegeneration, ChE inhibitors are still the most effective approach to the symptomatic treatment of neurodegenerative disorders. Recently, Kannan *et al*. [67] provided evidence of AChE inhibition by phloroglucinol and dibenzo [1,4] dioxine-2,4,7,9-tetraol and eckol, phlorotannins isolated from *Ecklonia maxima* (Osbeck) Papenfuss (from the West coast of South Africa) [67]. Both dibenzo [1,4] dioxine-2,4,7,9-tetraol and eckol proved to be stronger AChE inhibitors than phloroglucinol, probably because of greater molecular size and the presence of a larger number of hydroxyl groups, which are able to modulate the interaction with AChE and consequent inhibition of the enzyme [67].

**Figure 5:**
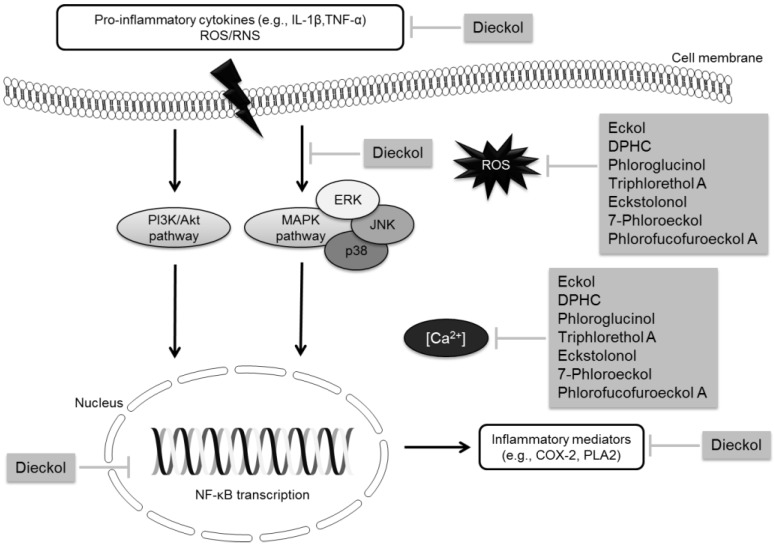
Polyphenols involved in neuroprotection mediated by anti-neuroinflammatory and antioxidant mechanisms.

**Figure 6:**
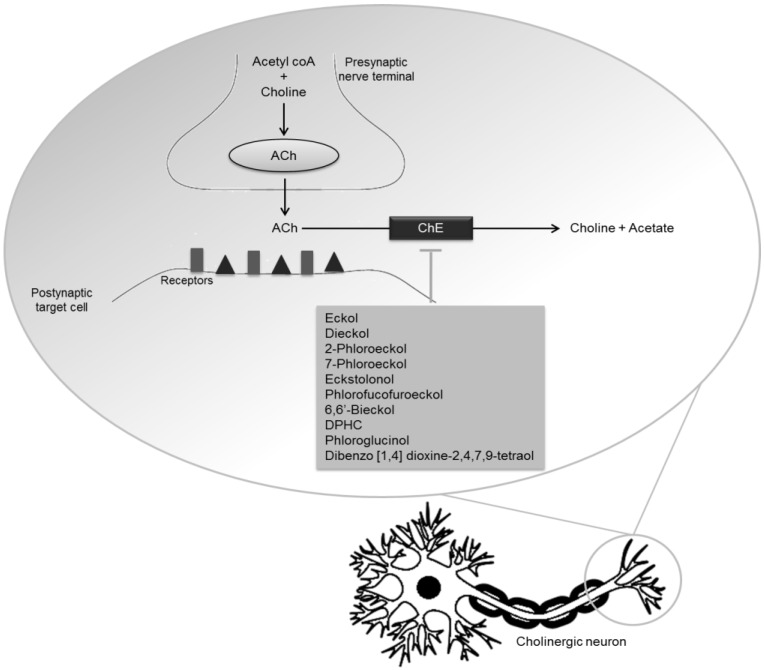
Polyphenols with ChE inhibitory activity.

These data suggest that macroalgae can be important sources of polyphenolic compounds with potential application as pharmaceutical or nutraceutical agents for prevention and control of neurodegenerative processes, through different pathways (Figure 5 and Figure 6).

### 3.2. Alkaloids

Alkaloids are heterocyclic nitrogen compounds, naturally occurring in plants, microbes, animals and marine organisms [68]. Alkaloids from marine algae are relatively rare, when compared with terrestrial plant alkaloids, and their biological potential is not fully known [69]. The relatively few alkaloids isolated from macroalgae can be distributed into four groups: 2-phenylethylamine, indole, halogenated indole and 2,7-naphthyridine derivatives. Alkaloids isolated from marine algae mostly belong to 2-phenylethylamine and indole groups. Halogenated alkaloids are specific for algae, being bromine- and chloride-containing alkaloids particularly dominant in Chlorophyta. Most of the alkaloids of the indole group are concentrated in Rhodophyta [70]. Some of these alkaloids exhibit numerous pharmacological effects like neuromodulation, neurotransmission, growth regulation, cytotoxicity, angiogenesis, antioxidant, as well as antibacterial, antifungal and larvicidal activities [71].

**Figure 7:**
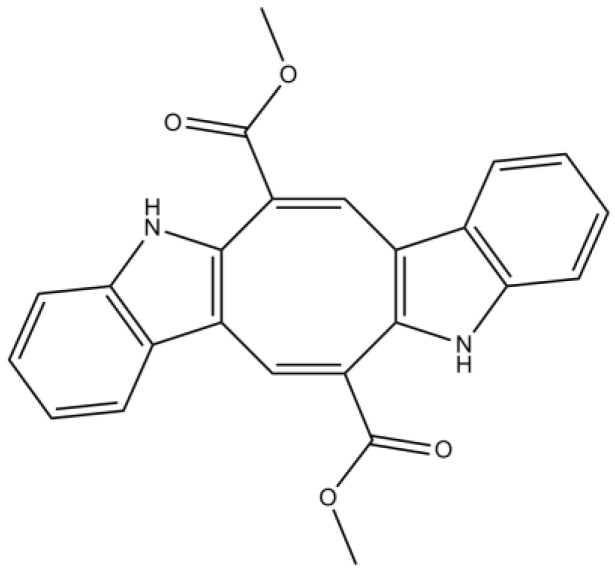
Chemical structure of caulerpin.

The indole alkaloids group comprises a large number of structurally diverse metabolites, such as bisindole alkaloids biosynthesized by a wide range of organisms. Different marine organisms, including macroalgae, provide numerous unique and intriguing bisindole alkaloids, which exhibit excellent bioactivities, thus attracting great interest from researchers [72,73]. De Souza *et al*. [74] have demonstrated that caulerpin (Figure 7), a bisindole alkaloid isolated from the lipoid extracts of *Caulerpa racemosa* (Forsskål) J. Agardh, collected in the Northeast of Brazil, was able to suppress inflammatory processes, probably as a result of an antioxidant effect and the inhibition of key enzymes involved in inflammation, such as cyclooxygenase (COX). The indole group of caulerpin is thought to be responsible for the prominent anti-inflammatory activity of this alkaloid [74].

**Figure 8:**
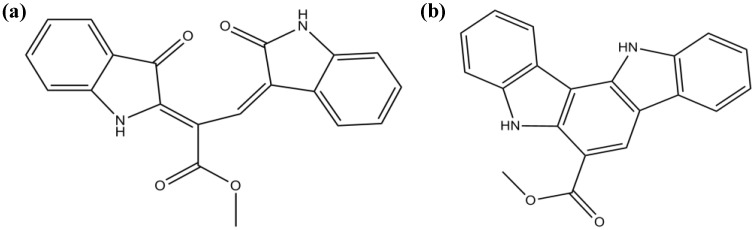
Chemical structure of racemosin A (**a**) and racemosin B (**b**).

Recently, two novel bisindole alkaloids, racemosins A and B, were isolated from *C. racemosa* (from the East China Sea) [73]. Racemosin A (Figure 8a) possesses a unique *seco*-indolo[3,2-α]carbazole skeleton with two indolinenone units, both conjugated with a methyl propenoate moiety, racemosin B (Figure 8b) being its cyclized derivative. Both alkaloids were evaluated *in vitro* for neuroprotective activity against Aβ-induced SH-SY5Y cell damage. The human neuroblastoma cell line SH-SY5Y is currently used in studies of neuroprotection as *in vitro*-simulated ischemia model. SH-SY5Y cells are generally known to be sensitive to oxidative stress and to express glutamate receptors [75]. The two compounds showed a certain degree of neuroprotection, racemosin A being the most powerful agent, which suggests that the distinctive scaffold of this particular alkaloid can be responsible for the modulation of important processes involved in neurodegeneration [73].

### 3.3. Terpenes

**Figure 9:**
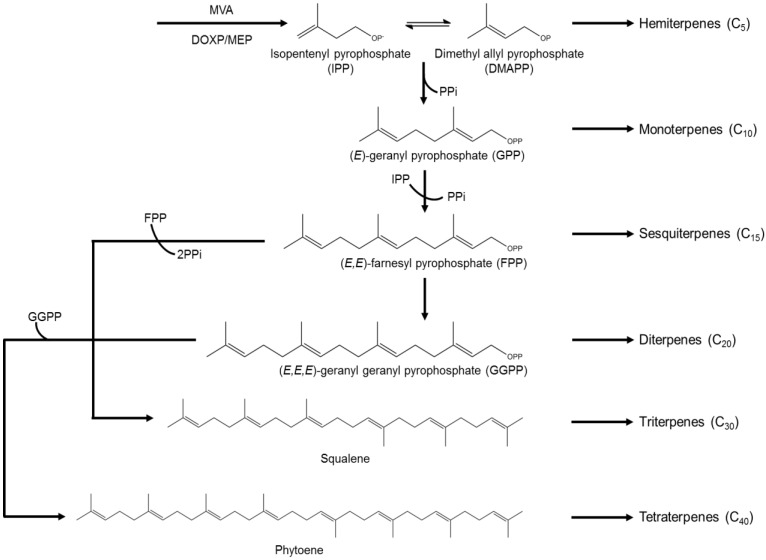
Schematic representation of the biosynthesis of the major subclasses of terpenes. Monoterpenes (C_10_), sesquiterpenes (C_15_) and diterpenes (C_20_) are derived from the corresponding intermediates, by sequential head-to-tail addition of C_5_ units. Triterpenes (C_30_) result from two C_15_ units linked head-to-head and tetraterpenes (C_40_) are formed from two C_20_ units, also joined head-to-head.

Terpenes represent one of the major classes of metabolites produced by marine algae [76]. Chemically, they can be considered to be derived from the five-carbon precursor isopentenyl pyrophosphate (IPP). Thus, terpenes are classified into hemiterpenes (C_5_), monoterpenes (C_10_), sesquiterpenes (C_15_), diterpenes (C_20_), sesterterpenes (C_25_), triterpenes (C_30_) and polyterpenes (>C_30_) (Figure 9) [77,78]. Biosynthetically, they are formed *via* two major pathways: mevalonate (MVA) and 1-deoxyxylulose 5-phosphate/2-C-methylerithrytol 4-phosphate (DOXP/MEP) [79]. Terpenes have shown promising biological potential, including anticancer, antioxidant, anti-inflammatory, antimicrobial activities, among others [79,80].

**Figure 10:**
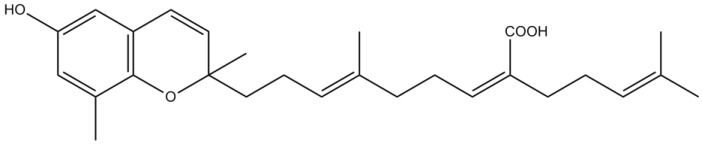
Chemical structure of sargachromenol.

Sargachromenol (Figure 10), a plastoquinone isolated from *Sargassum macrocarpum* C. Agardh, was shown to significantly promote nerve growth factor (NGF)-dependent neurogenesis in PC12 cells, in a dose-dependent manner [81]. PC12 cell line has been used as a model system to study the neuroprotective activity of neurotrophic factors, such as NGF, on neuronal cells. NGF has a crucial role in differentiation, survival and regeneration, by stimulating neuritis outgrowth in neuronal and rat phaeochromocytoma cells [82,83]. The neuroprotective effect exhibited by sargachromenol might be explained by the stabilization of microtubule assembling and extension of neuritis *via* protein kinase A (PKA) and MAPK signaling pathways [81].

**Figure 11:**
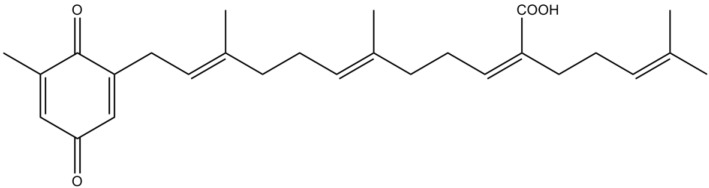
Chemical structure of sargaquinoic acid.

Kamei and Tsang [84] concluded that sargaquinoic acid (Figure 11), a quinonic compound isolated from *S. macrocarpum*, enhanced neuritis outgrowth in PC12D cells. The activation of both tyrosine kinase A (TrkA)-MAPK and adenylate cyclase-PKA proved to be essential mechanisms for the neurite outgrowth promoting effect of sargaquinoic acid [84]. Moreover, sargaquinoic acid was also able to mimic other neuroprotective effects of NGF, protecting PC12D cells from H_2_O_2_-induced oxidative stress. Although the molecular mechanism by which this antioxidant effect occurs is still not clear, the authors postulate that sargaquinoic acid may directly scavenge the free radical through its unsaturated isoprenoid moiety, or indirectly activate enzymes, such as catalase, to induce the free radical detoxifying mechanism [85].

Altogether, these results suggest that the use of sargachromenol and sargaquinoic acid as NGF-potentiating substances may regulate cellular responses, such as neuronal differentiation, neuroprotection and repair in the CNS [86].

More recently, Choi *et al*. [87] were able to isolate the two known meroterpenes, sargaquinoic acid and sargachomenol, from *Sargassum sagamianum* Yendo, collected along the coast of Jeju Island (Korea). Both compounds showed moderate AChE inhibitory activity; nevertheless, sargaquinoic acid was particularly potent against BuChE [87].

**Figure 12:**
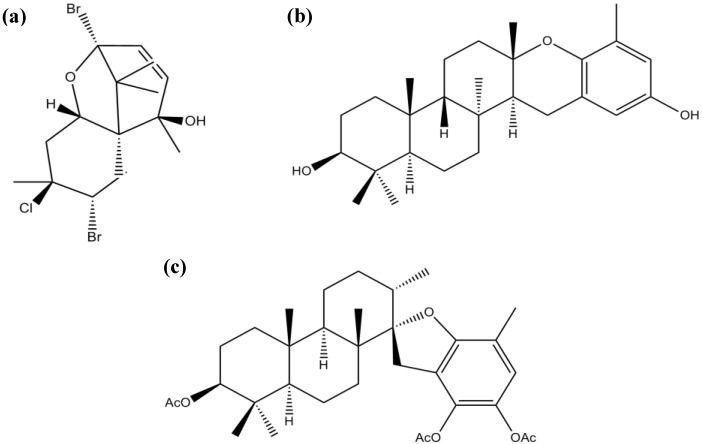
Chemical structure of pacifenol (**a**), epitaondiol (**b**) and stypotriol triacetate (**c**).

Pacifenol (Figure 12a), epitaondiol (Figure 12b) and stypotriol triacetate (Figure 12c) are terpenes found in different species of macroalgae. These compounds have been shown to exert a positive effect on inflammation through inhibition of the key enzyme phospholipase A2 (PLA2), which plays an important role in the release of arachidonic acid and formation of lipid mediators and consequent modulation of the COX pathway. Therefore, these compounds may be helpful in targeting processes that characterize many chronic inflammatory disorders, including AD and PD [88].

**Figure 13:**
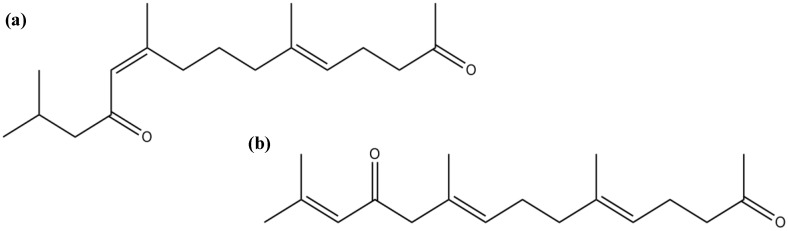
Chemical structure of (5*E*,10*Z*)-6,10,14-trimethylpentadeca-5,10-dien-2,12-dione (**a**) and (5*E*,9*E*,13*E*)-6,10,14-trimethylpentadeca-5,9,13-trien-2,12-dione (**b**).

Ryu *et al*. [89] isolated two farnesylacetone derivatives, (5*E*,10*Z*)-6,10,14-trimethylpentadeca-5,10-dien-2,12-dione (Figure 13a) and (5*E*,9*E*,13*E*)-6,10,14-trimethylpentadeca-5,9,13-trien-2,12-dione (Figure 13b), from the brown algae *S. sagamianum* (from Jeju Island, Korea), which showed moderate AChE and BuChE inhibitory activity [89]. The farnesylacetone derivatives share a similar skeleton with the two potent BuChE inhibitory plastoquinones previously described (sargachromenol and sargaquinoic acid). These data may suggest that the molecules’ scaffold represents an important feature in the modulation of the ChE inhibition.

**Figure 14:**
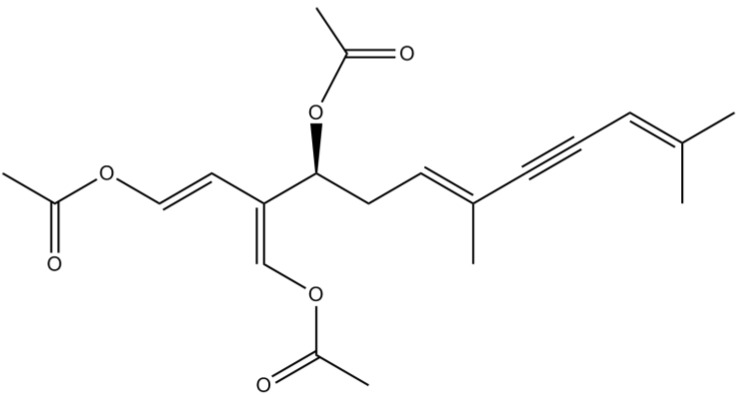
Chemical structure of caulerpenyne.

Caulerpenyne (Figure 14), a sesquiterpene isolated from the genus *Caulerpa* (species collected along the Turkish coastline), showed to be an effective lipoxygenase (LOX) inhibitor *in vitro* [90]. LOX is a family of iron-containing enzymes, which has proved to play an important role in neurodegenerative diseases through different mechanisms [91]. Previous reports have already demonstrated that LOX, particularly 5-LOX, an enzyme widely distributed within the CNS, is up-regulated in AD [92,93]. Therefore, LOX inhibitors like caulerpenyne could provide a novel therapeutic opportunity for AD.

Chang *et al*. [94] examined the neuroprotective activity of several terpenes *in vitro* using SH-SY5Y cells and developed a quantitative structure-activity relationship (QSAR) model for predicting the neuroprotective behavior of terpenes [94]. Indeed, QSAR models can contribute to reveal common proprieties, which govern the ligand–target interaction. In the future, this kind of tools can also be used for a better understanding of the neuroprotection capacity of terpenes and other classes of compounds isolated from macroalgae.

### 3.4. Pigments

Carotenoids, chlorophylls and phycobiliproteins are the main classes of pigments found in algae. Besides their photosynthetic and pigmentation effects, these compounds exhibit numerous beneficial health properties [95].

**Figure 15:**
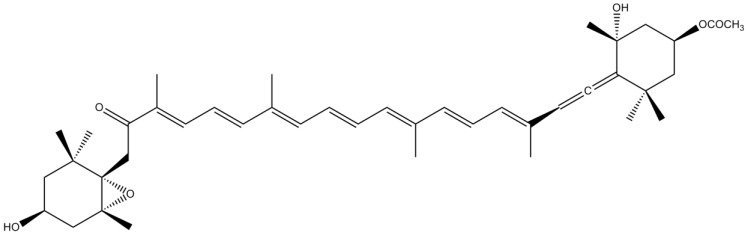
Chemical structure of fucoxanthin.

Carotenoids, the most widespread pigments in nature, derive from C_5_ isoprene units enzymatically polymerized to form regular, highly conjugated C_40_ structures (tetraterpenes) [96]. Carotenoids include two main subclasses: the nonpolar hydrocarbons carotenes, and the polar compounds xanthophylls [97]. Fucoxanthin is a xanthophyll with a unique structure, which includes an allenic bond and a 5,6-monoepoxide (Figure 15) [96]. Fucoxanthin is one of the most abundant carotenoids in nature and possesses numerous biological activities, including antioxidant, antitumor, anti-inflammatory, anti-obesity, anti-angiogenic and other interesting pharmacological effects [95,97].

Ikeda *et al*. [98] studied the effect of fucoxanthin isolated from *Undaria pinnatifida* (Harvey) Suringar (from a commercial cultivation ground in Japan), on neuronal cell death under ischemic conditions. They showed that fucoxanthin was able to attenuate neuronal cell damage in cortical neurons under hypoxia and re-oxygenation. Since ROS generation is considered to occur after hypoxia and oxygen reperfusion, it was assumed that the neuroprotective activity of fucoxanthin is mainly based on its scavenging activity [98]. The remarkable biological properties of fucoxanthin are based on its molecular structure: the presence of the allenic bond and intramolecular oxygen atoms may be correlated with the antioxidant activity [99,100].

Heo *et al*. [101] evaluated the cytoprotective effects of fucoxanthin isolated from *Sargassum siliquastrum* (Mertens ex Tuner) C. Agardh, collected along the coast of Jeju Island (Korea). The authors showed that fucoxanthin could effectively inhibit intracellular ROS formation, DNA damage and apoptosis induced by H_2_O_2_ [101].

More recently, it was shown that fucoxanthin suppresses indices of inflammation and oxidative damage in microglial cells, factors that have been implicated in the pathogenesis of neurodegenerative diseases. These effects were mediated *via* attenuation of the phosphorylation MAPK signaling pathway, as well as by the free radicals scavenging capacity of fucoxanthin and its ability to regulate the endogenous antioxidant system [102].

**Figure 16:**
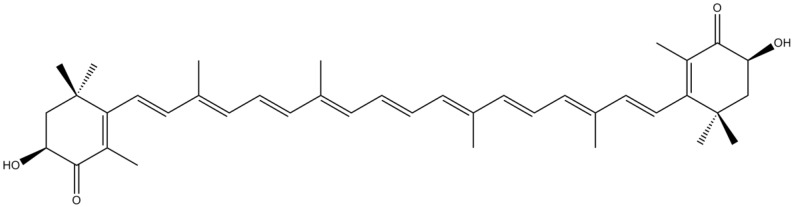
Chemical structure of astaxanthin (AST).

Astaxanthin (AST) is a xanthophyll found in a wide variety of living organisms, such as algae, salmon, trout, krill, shrimp and crayfish [103]. Its molecular structure is characterized by the presence of two terminal rings linked to a polyene chain (Figure 16). The unique and strong antioxidant properties exhibited by this pigment are deeply related to its many conjugated double bonds [104]. The neurotoxin 6-hydroxydopamine (6-OHDA) is a hydroxylated analog of dopamine usually employed in the study of dopaminergic degeneration, which presents a viable, but not completely accurate, model of PD [105]. Ikeda *et al.* [106] demonstrated that AST significantly suppressed 6-OHDA-induced apoptosis in SH-SY5Y cells *via* reduction of intracellular ROS generation, p38 MAPK pathway and mitochondrial dysfunctions [106]. Choi *et al.* [104] showed that AST was able to inhibit the production of inflammatory mediators by blocking the expression and formation of ^•^NO, inducible nitric oxide synthase (iNOS) and COX-2 [104]. AST also displayed neuroprotective effects against H_2_O_2_-induced toxicity *in vitro* and against cerebral ischemia *in vivo* [107,108]. The authors considered that these notable effects against brain damage were partly related to the strong antioxidant properties that characterize this pigment [107,108]. AST stabilizes free radicals by adding them to its polyene chain rather than donating an atom or electron to the radical. AST is, therefore, able to convert free radicals into more stable products and to terminate chain reactions. Also, the two hydroxyl groups in the 3 and 3′ positions render the molecules highly polar, allowing AST to span the cell membrane bilayer and consequently to enhance membrane functions [103,107]. Lu *et al.* [107] also hypothesized that the significant neuroprotective effect showed by AST could be also due to its anti-inflammatory activity [107]. Kim *et al.* [109] demonstrated that AST was able to reduce the expression of IL-6 *via* inhibition of MAPK signaling pathway in LPS-activated microglial cells [109]. Therefore, the anti-inflammatory activity exhibited by AST could be responsible for its neuroprotective effects. Moreover, Lee *et al.* [110] have shown that AST was able to protect SH-SY5Y cells and PD model mouse SN neurons from 1-methyl-4-phenylpyridinium (MPP^+^)/1-methyl-4-phenyl-1,2,3,6-tetrahydropyridine (MPTP)-induced mitochondrial dysfunction and ROS production through up-regulation of the expression of Bcl-2 protein, down-regulation of the expression of Bax and α-synuclein, and inhibition of the activation of caspase-3 [110]. More recently, Ye *et al.* [111] showed that AST exerted significant protective effects against MPP^+^-induced PC12 cells death through the suppression of ROS production and the inhibition of activated transcription factor 1 (Sp1)/NMDA receptor subunit 1 (NR1) signaling pathway [111]. Altogether, these results indicate that AST could provide a valuable therapeutic strategy for the treatment and/or prevention of neurodegenerative diseases such as AD and PD.

Chlorophylls are lipid-soluble pigments structurally characterized by the presence of a substituted tetrapyrrole with a centrally bound magnesium atom; the porphyrin tetrapyrrole is further esterified to a diterpene alcohol, phytol, to form chlorophyll [112]. Chlorophylls are sensitive to extreme pH and temperature conditions, allowing the formation of several derivatives, such as pheophytins [95].

**Figure 17:**
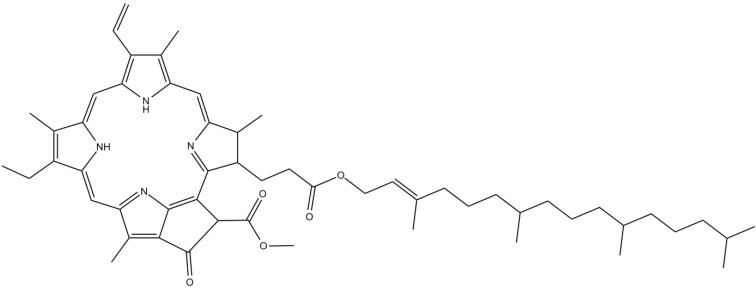
Chemical structure of pheophytin A.

Pheophytin A (Figure 17), a chlorophyll *a*-related substance found in many macroalgae species, was shown to be a strong neurodifferentiating compound. Ina *et al*. [113] demonstrated that pheophytin A isolated from *Sargassum fulvellum* (Turner) C. Agardh (from the Japanese coastline) was able to promote neuritis outgrowth in PC12 cells, through the activation of MAPK signaling pathway [113]. Neuritis outgrowth is a fundamental neuronal feature, playing an important role in neuronal development during embryogenesis and in the adult brain [114]. The key role of pheophytin A in neuritis outgrowth may be closely related to its low molecular weight [95].

More recent data demonstrated that pheophytin A isolated from the red algae *Odonthalia corymbifera* (S. G. Gmelin) Greville (collected at the coast of Hakodate, Japan) is a potent LOX inhibitor [115]. As already mentioned, LOX plays an important role in neurodegeneration. Therefore, LOX inhibitors like pheophytin A could provide a novel therapeutic opportunity for neurodegenerative diseases such as AD.

Phycobiliproteins (PBP) are one of the most important groups of proteins from algae. They generally comprise phycocyanins, allophycocyanins and phycoerythrins, the latter being the most abundant in Rhodophyta [95]. These water-soluble proteins are characterized by the presence of a tetrapyrrolic ring covalently attached to their structure [116]. Recent studies have shown that PBP, which generally make up 1%–10% of dry weight of algal biomass, impart antioxidant properties that could be beneficial in the prevention or treatment of several diseases associated with oxidative stress and inflammation [11].

To the best of our knowledge, only C-phycocyanin (C-PC) has been reported to have neuroprotective effects [117,118,119,120,121,122,123,124]. Several *in vitro* and *in vivo* studies have shown that C-PC was able to scavenge various radicals, such as alkoxyl, hydroxyl and peroxyl, and to inhibit lipid peroxidation, preventing oxidative damage [117,118,119,120,121,122]. C-PC was also able to protect SH-SY5Y cells from iron toxicity [123]. C-PC presumably exerted its effects by enhancing the activity of cellular antioxidant enzymes, such as glutathione peroxidase (GPx), glutathione reductase (GR) and a selenium-dependent glutathione peroxidase (GPx-Se), and also by increasing glutathione (GSH) levels in cells against oxidative stress induced by iron [123]. More recently, Pentón-Rol *et al.* [124] demonstrated that C-PC protected hippocampus neurons from death induced by global cerebral ischemia/reperfusion injury in gerbils. The authors suggested that the strong neuroprotective effect elicited by C-PC was presumably due not only to the reduction of ROS levels, but also to the possible inhibition of acute microglia activation [124].

These results indicate that algal pigments may represent novel and promising agents for treating or preventing several diseases related with oxidative damage and neuroinflammation.

### 3.5. Sterols

Sterols are abundant in macroalgae. These compounds can occur in the free form, esterified with fatty acids or be involved in glycosylated conjugates [125]. Algal sterols are extremely diverse and can be biosynthesized by two different pathways: MVA or DOXP⁄MEP [126]. The biosynthetic pathway depends on the evolutionary history, evidences showing that Chlorophyta exclusively use the DOXP⁄MEP pathway for sterol formation [127,128]. Sterols are amphipathic compounds with origin in isoprenoid biosynthesis, forming a group of triterpenes with a tetracyclic cyclopenta(α)phenanthrene structure and a side chain at C-17 [96]. Sterols from marine algae are structural and functionally similar to cholesterol; however, they contain an alkyl substitution at C-24 that is absent in cholesterol [129].

**Figure 18:**
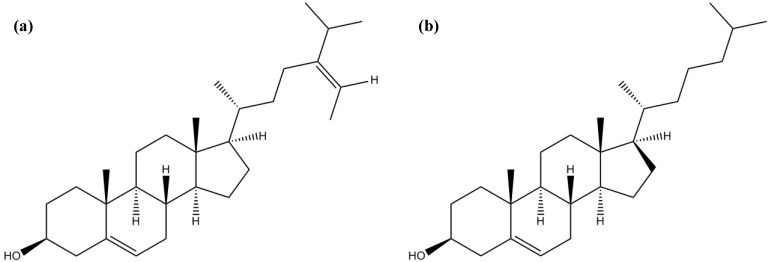
Chemical structure of fucosterol (**a**) and cholesterol (**b**).

Lopes *et al*. [126] found that fucosterol (Figure 18a) was the predominant sterol in Chlorophyta and Phaeophyta, while cholesterol (Figure 18b) was the main compound in Rhodophyta [126]. Yoon *et al*. [60] proved that fucosterol was a selective inhibitor of BuChE [60].

Andrade *et al.* [9] conducted the chemical and biological characterization of ethanolic extracts of 18 macroalgae species from the Portuguese coast [9]. In this study, a PCA analysis was performed allowing the establishment of a correlation between the algae chemical composition and the biological activity. Concerning ChE inhibition, Phaeophyta showed to be the most promising group, essentially due do the high amount of fucosterol present in some species, such as *Cystoseia usneoides* (Linnaeus) M. Roberts [9].

### 3.6. Oligo- and Polysaccharides

**Figure 19:**
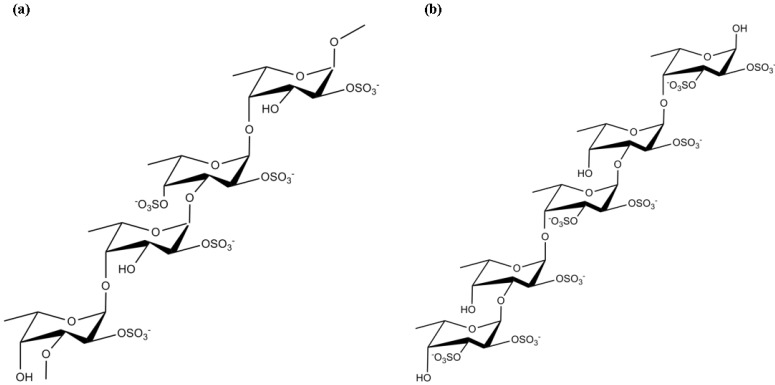
Chemical structure of fucoidan from *Fucus evanescens* C. Agardh (**a**) and from *F. vesiculosus* and *Ascophyllum nodosum* (Linnaeus) Le Jolis (**b**).

Fucoidan (Figure 19a,b) represents a class of sulfated polysaccharides extracted from brown algae. They are mainly composed of sulfated α-L-fucose residues, but may also contain galactose, mannose, xylose, uronic acids and acetyl groups [130]. Algal fucoidans exhibit numerous interesting biological activities, such as anticoagulant, anti-angiogenic [131], antiviral [132] and anti-inflammatory [31]. Due to the different chemical structure and composition, the biological effects of fucoidan proved to be dependent on the species from which it is isolated [131].

Within the context of neurodegenerative diseases, fucoidan was shown to protect Aβ-induced cholinergic neuronal death in rat models. Fucoidan pretreatment blocked the Aβ-induced ROS generation, as well as the activation of caspase-9 and caspase-3, which have been suggested to mediate the terminal stages of neuronal apoptosis [130].

Luo *et al*. [133] demonstrated that fucoidan obtained from *Laminaria japonica* Areschoug, (commercially cultured in China), significantly improved locomotor activity and was also able to protect against depletion of striatal dopamine *in vivo*. The authors suggested that the protective effect of fucoidan in MPTP-induced neurotoxicity models could be partly related to its antioxidant action [133]. Other *in vitro* and *in vivo* studies showed that fucoidan was able to suppress the production of pro-inflammatory factors in lipopolysaccharide (LPS)-activated microglial cells, possibly mediated by the down-regulation of the MAPK signaling pathway. Therefore, the neuroprotective effects of fucoidan were suggested to be explained by its anti-inflammatory activity [31,134,135].

In a study conducted by Gao *et al*. [136] fucoidan was able to protect PC12 cells against H_2_O_2_-induced apoptosis *via* reduction of ROS levels and activation of phosphatidylinositol-3-kinase (PI3K)/Akt survival pathway [136].

Altogether, these data suggest that fucoidan may be a therapeutic agent for the treatment of inflammatory conditions, including neurodegenerative diseases. Nevertheless, it would be important to fully characterize the chemical features of fucoidan molecules in order to distinguish or establish reliable correlations between structures and specific biological activities of fucoidans.

**Figure 20:**
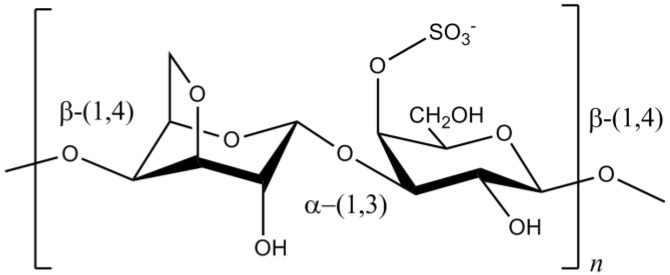
Chemical structure of κ-carrageenan.

Carrageenans are sulfated polysaccharides commonly extracted from Rhodophyta. The commercially important κ-carrageenan contains a 3,6-anhydro-α-d-galactopyranose, which is responsible for its gelling properties (Figure 20) [137]. Besides this, κ-carrageenan has exhibited prominent biological effects, including antitumor [138], antioxidant [139] and anti-inflammatory [140]. Regarding to neurodegenerative diseases, studies have already showed that κ-carrageenan was able to protect microglial cells against LPS toxicity through the reduction of the viability and content of ^•^NO, TNF-α and IL-10 [140,141]. The authors suggested that the protective function of κ-carrageenan was positively correlated to the sulfate group content of the studied oligosaccharides [140,141]. Therefore, κ-carrageenan can potentially be used for preventing the neurodegenerative processes of some CNS diseases.

### 3.7. Fatty Acids

**Figure 21:**
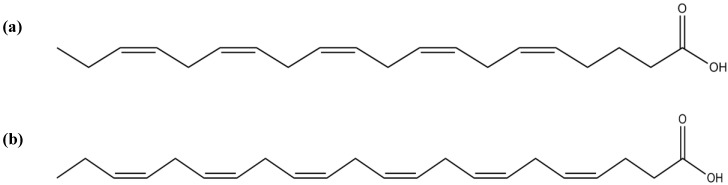
Chemical structure of EPA (**a**) and DHA (**b**).

Macroalgae are rich in polyunsaturated fatty acids (PUFAs), mainly eicosapentaenoic (EPA; 20:5*n*-3) (Figure 21a) and docosahexaenoic (DHA; 22:6*n*-3) acids (Figure 21b) [142]. EPA and DHA are considered to be the two most important PUFAs of marine lipids, since they are intricately related to important biological effects, such as cardiovascular protection, anti-inflammatory and anticancer [143,144,145].

There is growing evidence that essential fatty acids’ deficiency is an important risk factor for AD and PD [146,147,148]. Indeed, several epidemiological studies and studies conducted in animals highlighted the beneficial effect of PUFAS, particularly DHA, against neurodegenerative diseases [148]. PUFAs are primary components of the brain and are related to membrane integrity and fluidity [149]. DHA is the predominant omega-3 PUFA, not only in the brain, but also in the retina and an adequate supply of DHA is essential for proper brain, eye, and nerve functions. The main source of DHA is our diet [150]. For this reason, the use of exogenous DHA or other PUFA could stand up as nutraceutical defenses against brain diseases. Farooqui and Horrocks [149] have reported that reduced brain levels of DHA could be responsible for abnormal signal transduction associated with learning disability and cognitive deficit [149]. More recently, Tracy *et al*. [151] have also demonstrated that exposure to saturated fatty acids (SFA) can promote and strengthen some effects of inflammatory triggers on microglial cells [151]. Therefore, low intake of PUFAs and high consumption of SFA may increase the risk of developing certain forms of dementia [143].

Lipid replacement therapy (LRT) has been used together with other strategies, such as antioxidant therapy, in order to replace damaged or oxidized lipids present in cellular and organelle membranes, which are usually accumulated during aging and in numerous clinical conditions [152,153]. Although not every clinical study has found health benefits from lipid dietary supplementation, most studies have reported the value of certain types of lipids, such as *n*-3 PUFAs [154].

Across the years, several studies have provided important results concerning fatty acids profiles of numerous macroalgae species. Generally, they showed that macroalgae, especially Phaeophyta and Rhodophyta species, are valuable sources of PUFAs [145,155,156,157,158]. The levels and the proportions of PUFAs algal lipids vary depending upon the species, the geographical origin and the exposure to diverse abiotic factors (e.g., temperature) [156,157,158].

Regarding to neurodegenerative diseases, Ren *et al*. [159] reported that PUFAs have a relatively moderate effect on AChE, DHA being the strongest inhibitor. The authors suggested that long-chain PUFAs possess specific molecular features responsible for this promising activity [159]. Andrade *et al*. [9] were able to identify and quantify eight fatty acids from 18 species of macroalgae, collected along the Portuguese coastline. They showed that fatty acids were the most represented metabolites, both in diversity and content. Among the fatty acids identified it was suggested that EPA, oleic and arachidonic acids could be involved in AChE inhibition [9].

Several mechanisms have been proposed to explain the protective role of PUFAs in neurological disorders. Akbar *et al*. [160] demonstrated that DHA promoted neuronal survival by the maintenance of basal Akt activity [160]. DHA also increased cortical brain derived neurotrophic factor (BDNTF) [161] and its ethanolamide metabolites showed to promote neuritis growth and synaptogenesis [162]. Mukherjee *et al*. [163] demonstrated that 10,17*S*-docosatriene (neuroprotectin D1 (NPD1)), a stereospecific DHA-derived mediator was able to modulate specific signaling pathways that promote cell survival. Overall, NPD1 protected retinal pigment epithelium cells from oxidative-stress-induced apoptosis. The authors predict that these effects would similarly protect neuronal cells. NPD1 was able to up-regulate the anti-apoptotic proteins Bcl-2 and Bcl-x_L_ decreasing pro-apoptotic Bax and Bad expression. Moreover, NPD1 inhibited oxidative-stress-induced caspase-3 activation and IL-1-stimulated expression of COX-2 [163].

More recently, Orr *et al*. [164] also showed that unesterified DHA provided protection in LPS-induced mouse model of acute neuroinflammation [164].

Although EPA usually occurs at low to nearly non-detectable levels in neural tissue, EPA-derived product resolvin E1, as well as 5- and 18-hydroxy-EPA, were detected in the hippocampus. Since these metabolites possess anti-inflammatory properties in non-neural tissues, it is possible that they could also play a determinant role in brain inflammation, being crucial to exploit their potential in future research [164,165].

These data suggest that PUFAs present in macroalgae may provide novel approaches for the prevention and treatment of neurological disorders with a neuroinflammatory component.

### 3.8. Other Compounds

**Figure 22:**
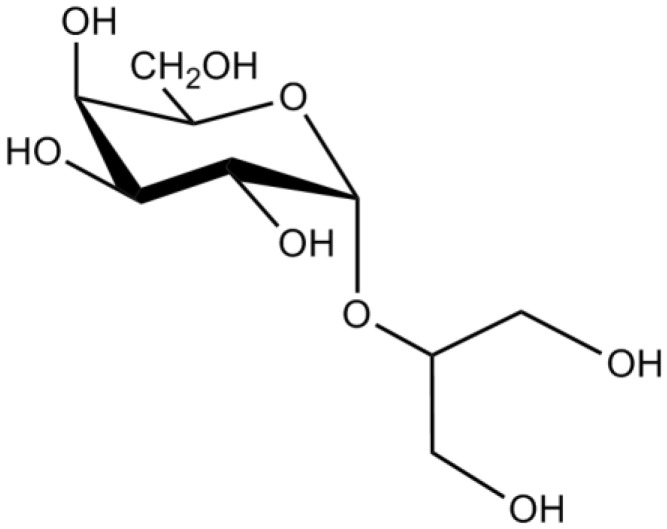
Chemical structure of floridoside.

Floridoside (2-*O*-d-glycerol-*α*-d-galactopyranoside), a natural glycerol glycoside, is the main photosynthetic product of many Rhodophyta (Figure 22). Recent studies have shown that floridoside was able to suppress the neuroinflammatory response of LPS-activated microglial cells and the subsequent production of ROS and ^•^NO, probably by the inhibition of MAPK signaling pathway. The authors suggested that floridoside could be a viable therapeutic agent against neuroinflammation-mediated neurodegeneration [32].

## 4. Conclusions 

Macroalgae are prominent and potentially renewable sources of compounds with a vast array of bioactivities, including neuroprotective effects.

Numerous compounds isolated from macroalgae have shown to exert neuroprotective effects through a multiplicity of mechanisms, which are summarized in Table 1.

**Table 1:** Neuroprotective compounds from macroalgae and possible mechanisms involved.

Class	Compound		Neuroprotective Effects	References
**Simple phenol**	Phloroglucinol		Suppression of the overproduction of intracellular ROS, decrease of intracellular Ca^2+ ^levels and reduction of apoptosis	[35]
**Phlorotannins**	Dieckol		Inhibitory activity against AChE	[67]
			Reduction of the expression and release of ^•^NO, PGE2, IL-1β and TNF-α in microglial cells	[29]
			Suppression of the overproduction of intracellular ROS, decrease of intracellular Ca^2+ ^levels and reduction of apoptosis	[35]
	Phlorofucofuroeckol		Inhibitory activity against AChE	[59]
	Phlorofucofuroeckol A		Inhibitory activity against AChE and BuChE	[60]
			Suppression of intracellular ROS generation and decrease of Ca^2+^ levels	[62]
	Eckol		Inhibitory activity against AChE	[60,67]
			Suppression of the overproduction of intracellular ROS, decrease of intracellular Ca^2+ ^levels and reduction of apoptosis	[35]
	2-Phloroeckol		Inhibitory activity against AChE	[60]
	7-Phloroeckol		Inhibitory activity against AChE	[60]
			Suppression of intracellular ROS generation and decrease of Ca^2+^ levels	[62]
**Phlorotannins**	Eckstolonol		Inhibitory activity against AChE and BuChE	[60]
			Suppression of the overproduction of intracellular ROS, decrease of intracellular Ca^2+ ^levels and reduction of apoptosis	[35]
	6,6′-Bieckol		Potent inhibitory activity against AChE (non-competitive inhibition type)	[61]
	DPHC		Moderate inhibitory activity against BuChE	[61]
			Antioxidant mechanisms and control of intracellular Ca^2+^ levels	[38]
	Triphlorethol A		Suppression of the overproduction of intracellular ROS, decrease of intracellular Ca^2+ ^levels and reduction of apoptosis	[35]
	Fucophlorethol A		Scavenging of reactive carbonyls, inhibiting the formation of AGEs	[66]
	Tetrafucol A		Scavenging of reactive carbonyls, inhibiting the formation of AGEs	[66]
	Trifucodiphlorethol A		Scavenging of reactive carbonyls, inhibiting the formation of AGEs	[66]
	Dibenzo [1,4] dioxine-2,4,7,9-tetraol		Inhibitory activity against AChE	[67]
**Alkaloids**	Caulerpin		Antioxidant properties	[74]
			Moderate/weak attenuation of Aβ-induced SH-SY5Y cell damage (unknown mechanism)	[73]
	Racemosin A		Strong attenuation of Aβ-induced SH-SY5Y cell damage (unknown mechanism)	[73]
	Racemosin B		Moderate/weak attenuation of the Aβ-induced SH-SY5Y cell damage (unknown mechanism)	[73]
**Terpenes**	**Meroterpenes**	Sargachromenol	Promotion of NGF–dependent neurogenesis by stabilization of the microtubule assembling and extension of neuritis *via* PKA and MAPK signaling pathways	[81]
			Moderate inhibitory activity against AChE	[87]
		Sargaquinoic acid	Enhancement of neuritis outgrowth *via* TrkA‑MAPK and adenylate cyclase‑PKA signaling pathways	[84]
			Antioxidant properties	[85]
			Potent inhibitory activity against BuChE and moderate inhibitory activity against AChE	[87]
	**Meroditerpenes**	Epitaondiol	Inhibition of PLA2 and COX pathway	[88]
		Stypotriol triacetate		
	**Sesquiterpenes**	Pacifenol	Inhibition of PLA2 and COX pathway	[88]
		(5*E*,10*Z*)-6,10,14-trimethylpentadeca-5,10-dien-2,12-dione	Moderate inhibitory activity against AChE and BuChE	[89]
		(5*E*,9*E*,13*E*)-6,10,14-trimethylpentadeca-5,9,13-trien-2,12-dione		
		Caulerpenyne	Inhibitory activity against LOX	[90]
**Carotenoids**		Fucoxanthin	Attenuation of neuronal cell damage through scavenging activity	[98]
			Inhibition of intracellular ROS formation, DNA damage, and apoptosis induced by H_2_O_2_	[101]
			Suppression of inflammation and oxidative damage in microglial cells *via* inhibition of MAPK pathway	[102]
**Carotenoids**	AST		Suppression of expression and formation of ^•^NO, iNOS and COX-2	[104]
			Suppression of intracellular ROS generation, mitochondrial dysfunctions and p38 MAPK pathway	[105]
			Antioxidant properties	[107,108]
			Reduction of the expression of IL-6 *via* inhibition of MAPK signaling pathway	[109]
			Suppression of MPP^+^/MPTP-induced mitochondrial dysfunction and ROS production *via* up-regulation of the expression of Bcl-2 protein, down-regulation of the expression of Bax and α-synuclein, and inhibition of the activation of caspase-3	[110]
			Suppression of ROS production and inhibition of Sp1/NR1 signaling pathway	[111]
**Chlorophyll derivatives**	Pheophytin A		Enhancement of neuritis outgrowth *via* the activation of MAPK pathway	[113]
			Potent inhibitory activity against LOX enzymes	[115]
**Sterols**	Fucosterol		Moderate inhibitory activity against BuChE	[60]
			Inhibitory activity against AChE and BuChE	[9]
**Phycobiliproteins**	C-PC		Scavenge of numerous radicals and inhibition of lipid peroxidation, preventing oxidative damage	[117,118,119,120,121,122]
			Protection against iron-induced SH-SY5Y toxicity through the increase of cellular antioxidant enzymes (GPx, GR, GPx-Se) and GSH levels	[123]
			Protection of hippocampus neurons induced by global cerebral ischemia/reperfusion injury through the reduction of ROS levels and possible inhibition of acute microglia activation	[124]
**Oligo- and Polysaccharides**	Fucoidan		Suppression of ROS generation and activation of caspase-9 and caspase-3	[130]
			Antioxidant properties	[133]
			Activation of PI3K/Akt survival pathway	[136]
	κ-carrageenan		Inhibition of the viability and content of ^•^NO, TNF-α and IL-10 released by LPS-activated microglia cells	[139,141]
**Fatty acids**	EPA		Moderate inhibitory activity against AChE	[9,159]
			Anti-inflammatory activity of EPA-derived products (resolvin E1, and 5- and 18-hydroxy-EPA)	[165]
	DHA		Inhibitory activity against AChE	[9,159]
			Promotion of neuronal survival by positive modulation of Akt	[160]
			Enhancement of neuritis growth and synaptogenesis by DHA ethanolamide metabolites	[162]
			DHA-derived mediator (NPD 1) promotes cell survival presumably through the up-regulation of Bcl-2 and Bcl-x_L_, down-regulation of Bax and Bad, suppression of oxidative stress-induced caspase-3 activation and IL-1-stimulated expression of COX-2	[163]
**Glycerol glycosides**	Floridoside		Suppression of pro-inflammatory responses in microglia through the inhibition of the production of ^•^NO and ROS and blockage of MAPK pathway	[32]

Neurodegenerative diseases are characterized by complex and deeply related phenomena, such as neuroinflammation, oxidative/nitrosative damage and synaptic loss. Therefore, some of the compounds described in this review can represent viable alternatives in the management of neurodegenerative diseases like Alzheimer’s and Parkinson’s.

According to the neuroinflammatory hypothesis of neurodegenerative diseases, compounds exerting anti-inflammatory effects should slow the disease progression. Dieckol, caulerpin, pacifenol, epitaondiol, stypotriol triacetate, fucoxanthin, pheophytin A, fucoidan, κ-carrageenan, floridoside, and PUFAs were shown to suppress inflammatory responses mostly by antioxidant effects and *via* regulation of different signaling pathways. Several studies have also established that the imbalance between pro-oxidant and antioxidant homeostasis may be involved in the pathogenesis of neurodegenerative diseases. Phlorotannins, sulfated polysaccharides, carotenoids and sterols isolated from different macroalgae species showed potent antioxidant properties. Nevertheless, the application of ChE inhibitors in symptomatic treatment of neurodegenerative diseases still appears to be the most effective approach. Many phlorotannins, as well as sargachromenol, sargaquinoic acid and PUFAs, exhibited inhibitory activities against AChE and/or BuChE. ChE inhibitors are frequently related to a number of side effects; therefore, it can be suggested that the employment of compounds extracted from macroalgae instead of synthetic ingredients could reduce the risk of toxicity in the organism and prevent the occurrence of certain undesirable effects.

Despite the growing evidences that compounds from marine resources, particularly macroalgae, may constitute valuable therapeutic candidates for neurodegenerative diseases, few of them will be successfully marketed with the current technologies available [105]. The supply of macroalgae required to conduct preclinical and clinical trials can markedly affect the marine ecosystem’s sustainability. Therefore, novel and greener strategies are necessary to overcome the supply problem. Besides, the composition in bioactive compounds strongly varies among species, being affected by numerous factors, such as algae size, age, tissue type, salinity, season, nutrient levels, intensity of herbivory, light intensity and water temperature [52]. In this sense, it is also crucial to implement efficient cultivation techniques, which could not only prevent the overexploitation of natural populations, but also facilitate the development and propagation of genotypes in order to produce high value chemicals for potential application in the pharmaceutical and nutraceutical sectors [166].

Altogether, the data reviewed demonstrate that numerous compounds isolated from different species of macroalgae can be used as neuroprotective agents. Nevertheless, further research is needed in order to explore their maximum therapeutic potential, for novel and successful application as pharmaceuticals and nutraceuticals for the treatment and/or prevention of neurodegenerative diseases.
